# Cardiac diastolic maladaptation is associated with the severity of exercise intolerance in sickle cell anemia patients

**DOI:** 10.1038/s41598-024-61689-w

**Published:** 2024-05-15

**Authors:** Thomas d’Humières, Antoine Bouvarel, Laurent Boyer, Laurent Savale, Henri Guillet, Lara Alassaad, Gonzalo de Luna, Enora Berti, Sihem Iles, Anne Laure Pham Hung d’Alexandry d’Orengiani, Etienne Audureau, Marie-Joelle Troupe, Reine-Claude Schlatter, Anaïs Lamadieu, Frédéric Galactéros, Geneviève Derumeaux, Laurent A. Messonnier, Pablo Bartolucci

**Affiliations:** 1 Physiology Department, FHU SENEC, Henri Mondor Hospital, Assistance Publique Hôpitaux de Paris (AP-HP), Créteil, France; 2 INSERM IMRB U955, Université Paris Est (UPEC), 51 Avenue du Maréchal de Lattre de Tassigny, 94010 Créteil, France; 3 Department of Respiratory and Intensive Care Medicine, Pulmonary Hypertension National Referral Center, Hôpital Bicêtre, Assistance Publique Hôpitaux de Paris (AP-HP), Le Kremlin-Bicêtre, France; 4 School of Medicine, Université Paris-Saclay, Le Kremlin-Bicêtre, France; 5 Department of Internal Medicine, Henri-Mondor University Hospital-UPEC/Assistance Publique-Hôpitaux de Paris (AP-HP), Créteil, France; 6 Sickle Cell Referral Center-UMGGR, Plateforme d’expertise Maladies Rares Grand Paris Est, UPEC, FHU SENEC, CHU Henri Mondor APHP, Créteil, France; 7 Biostatistics Department, Henri Mondor Hospital, Assistance Publique Hôpitaux de Paris (AP-HP), Créteil, France; 8 CEpiA IMRB U955, FHU SENEC, Université Paris Est (UPEC), Créteil, France; 9 INSERM UMR_S 999 Pulmonary Hypertension: Pathophysiology and Novel Therapies, Hôpital Marie Lannelongue, Le Plessis-Robinson, France; 10 Inter-University Laboratory of Human Movement Sciences EA 7424, Université Savoie Mont Blanc, Chambéry, France

**Keywords:** Genetics, Cardiology, Medical research, Haematological diseases, Physiology, Metabolism

## Abstract

This pilot study focusing on Sickle Cell Anemia (SCA) patients offers a comprehensive and integrative evaluation of respiratory, cardiovascular, hemodynamic, and metabolic variables during exercise. Knowing that diastolic dysfunction is frequent in this population, we hypothesize that a lack of cardiac adaptation through exercise might lead to premature increase in blood lactate concentrations in SCA patients, a potential trigger for acute disease complication. SCA patients were prospectively included in PHYSIO-EXDRE study and underwent a comprehensive stress test with a standardized incremental exercise protocol up to 4 mmol L^−1^ blood lactate concentration (BL4). Gas exchange, capillary lactate concentration and echocardiography were performed at baseline, during stress test (at ∼ 2 mmol L^−1^) and BL4. The population was divided into two groups and compared according to the median value of percentage of theoretical peak oxygen uptake (%${\dot{\text{V}}\text{O}}_{2peakth}$) at BL4. Twenty-nine patients were included (42 ± 12 years old, 48% of women). Most patients reached BL4 at low-intensity exercise [median value of predicted power output (W) was 37%], which corresponds to daily life activities. The median value of %${\dot{\text{V}}\text{O}}_{2peakth}$ at BL4 was 39%. Interestingly, diastolic maladaptation using echocardiography during stress test along with hemoglobin concentration were independently associated to early occurrence of BL4. As BL4 occurs for low-intensity exercises, SCA patients may be subject to acidosis-related complications even during their daily life activities. Beyond assessing physical capacities, our study underlines that diastolic maladaptation during exercise is associated with an early increase in blood lactate concentration.

## Introduction

Sickle cell disease (SCD) is the most common inherited blood disorder in the world^1^. Poor physical capacities are common in patients homozygous for HbS—also called sickle cell anemia (SCA)—as a result of anemia and multiple pulmonary, cardiac, endothelial, muscle, and metabolic dysfunctions^2^. For these patients, elevated pulmonary arterial pressure, diastolic dysfunction, impaired oxygen transport and delivery^3^ and early blood lactate accumulation^4,5^ translate into poor adaptation to physical activities. During an exercise of increasing intensity, significant activation of glycogenolysis and glycolysis leads to muscle lactate production, accumulation and subsequently to an increase in blood concentrations^6,7^. These exercise-related metabolic changes result in a left shift of the blood lactate (vs. work load) curve, with premature occurrence of the first lactate threshold and of the 4 mmol L^−1^ blood lactate concentration (BL4). Furthermore, elevation of blood lactate concentrations is accompanied by acidosis^8^.

Regarding SCA patients, acidosis is well-known for promoting hemoglobin S (HbS) deoxygenation, increasing the risk of polymerization, potentially inducing red blood cell sickling, and ultimately leading to vaso-occlusive crisis and hemolysis^9^. Therefore, an in-depth analysis of those patients during exercise is crucial to better understand the link between metabolic status and cardiovascular adaptation. Indeed, cardiovascular abnormalities at rest, such as pulmonary hypertension (PH) and diastolic dysfunction, have been identified as the leading cause of mortality and impaired quality of life in SCA patients^10–14^. These disorders rely on complex mechanisms associating chronic anemia, hemolysis-induced vasculopathy and repeated vaso-occlusive episodes, contributing to a maladaptive cardiovascular remodeling^15–17^. While the diagnosis of cardiac dysfunction is extremely challenging considering the unique hemodynamic features of SCA, recent studies have reported the potential interest of investigating cardiovascular adaptation during exercise, to dynamically unmask latent heart dysfunction and its association with exercise limitation^18–20^. However, there is currently no data available on the link between cardiac impairment, premature lactate accumulation and thus the poor physical capacities of SCA patients during stress test.

This pilot study is designed to comprehensively evaluate multiple exercise parameters by integrating cardiovascular, hemodynamic, respiratory, and metabolic variables. The main hypothesis of this work is that subclinical cardiac diastolic alterations during exercise might lead to premature increase in blood lactate concentrations, assessed by percentage of theoretical peak oxygen uptake (%${\dot{\text{V}}\text{O}}_{2peakth}$) at BL4. The secondary objective was to evaluate the safety of the procedure.

## Methods

### Study design and patients

This pilot study was carried out in collaboration between the Physiology Department and the Sickle Cell Disease Referral Center, at Henri Mondor University Hospital, Creteil, France. Consecutive SCA adult patients referred by the SCD Referral Center for a cardiovascular pre-rehabilitation assessment were approached to participate in this prospective exploratory study from September 2020 to July 2021. SCA patients ≥ 18 years of age, at steady state condition and with health insurance coverage. Patients under administration of beta-blockers or other bradycardic therapies, or with specific SCD treatments (erythropheresis, hydroxyurea, blood transfusion) were also included. Exclusion criteria were (i) a contraindication for or inability to cycling on ergometer, (ii) patients under guardianship, curatorship, or legal protection, and (iii) pregnant or nursing women. This study was performed in line with the principles of the Declaration of Helsinki. All patients provided written informed consent. The protocol was supervised by a data safety monitoring board and the trial was approved by the ad hoc ethics committee (Comité de Protection des Personnes Ouest III; APHP 201322; EudraCT ID RCB 2020-A02842-37).

### Research hypothesis

Exercise intolerance is multifactorial in SCA patients. Through a comprehensive and standardized evaluation of parameters associated with early anaerobic shift, we though to highlight cardiac function adaptation through exercise as a pathophysiological pathway associated with early blood lactates accumulation, ultimately leading to a reduced workload.

### Data collection

All participants were interviewed, underwent physical examination and their medical records were examined. Height (cm) and body mass (kg) were recorded, and body mass index was calculated. History of past complications of SCD was documented, including vaso-occlusive crisis, acute chest syndrome (ACS), priapism, skin ulcers, osteonecrosis, chronic organ damage [pulmonary hypertension (PH) documented on right heart catheterization (RHC), cerebral vasculopathy, renal disease, retinopathy] and in intensive care units. Laboratory tests performed in the SCD Referral Center (complete blood count, serum chemistry profile, and lactate dehydrogenase less than a month before the procedure) were collected.

### Procedure

**Figure 1:**
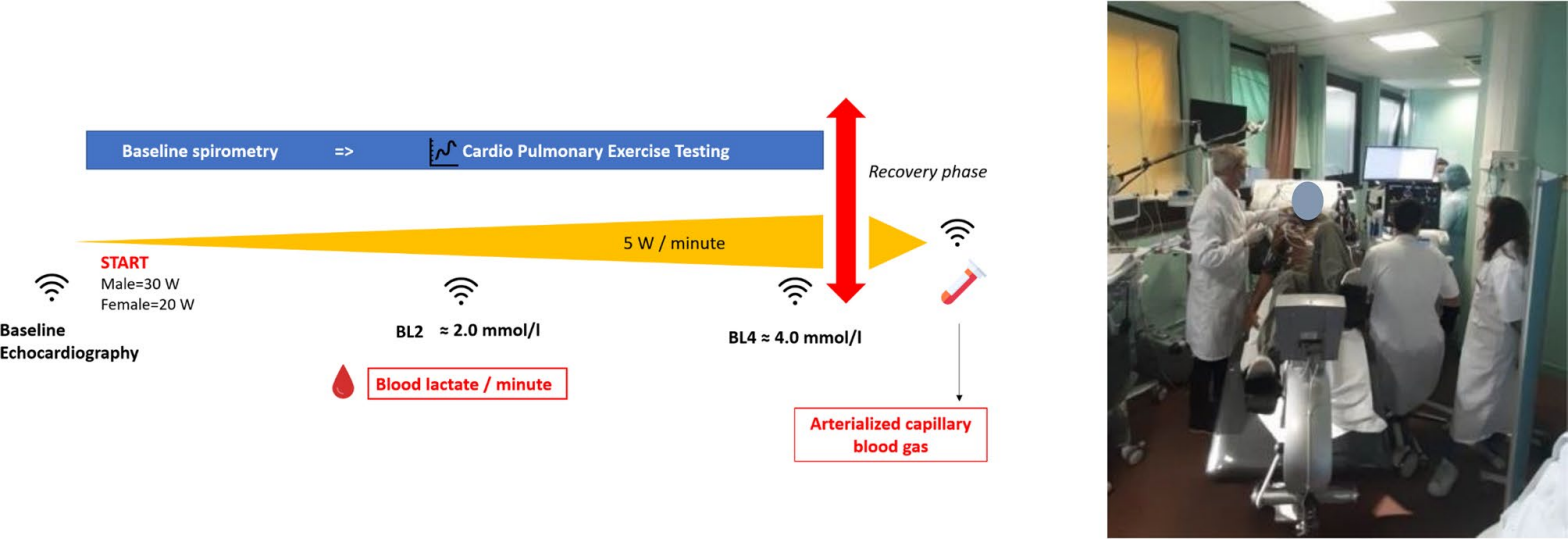
Experimental protocol.

All patients underwent the same single session of examination in the Physiology Department (altitude + 50 m), Fig. 1. Investigators led by a cardiologist were blinded to patients’ history. Firstly, patients underwent an echocardiography (VIVID E95—General Electric, Boston, USA) at rest. All patients performed a baseline spirometry to determine the forced expiratory volume in 1 s (FEV1) and the forced vital capacity (FVC). Then, a hyperhemic cold cream (Dolpic, Montbonnot, France) was applied onto the earlobe and removed right before the exercise. After, patients performed a submaximal cardiopulmonary exercise test (CPET) on a stationary electronic cycle ergometer (Koch Evolution-Tech, Le Haillan, France) as previously proposed^2^. Patients started CPET at a power output of 20 or 30 W (for women and men, respectively), with an increase of 5 W every minute. Exercise was stopped as soon as blood lactate reached a concentration ≥ 4 mmol L^−1^. At rest and every minute during exercise, samples of 10 µL of arterialized capillary blood were collected from the earlobe and analyzed extemporaneously within 15–20 s (EKF lactate scout 4 reader, Cardiff, United-kingdom) to determine whole blood lactate concentration ([lactate]_b_, mmol L^−1^) and establish a blood lactate (vs. work rate) curve for each patient. Stress echocardiography data (VIVID E95—General Electric, Boston, USA) were collected when lactate concentrations reached ∼ 2 mmol L^−1^ (BL2, which can be interpreted as the first lactic threshold^21^) and ∼ 4 mmol L^−1^ (BL4) as well as during the early phase (within 2 min) of recovery (procedure detailed below and in Fig. 1). Two minutes after exercise completion, a blood sample of 80 µL was withdrawn from the earlobe to measure blood gas including pH (ABL90 Flex, Radiometer, København, Denmark). Blood pressure was measured automatically every 2 min, while pulse oximetry (peripheral oxygen saturation), 12-lead electrocardiogram, heart rate and gas exchange were continuously monitored. Gas exchange at rest, during exercise and in recovery were analyzed to determine minute ventilation (${\dot{{\text{V}}}{\text{E}}}$, L min^−1^), oxygen uptake (${\dot{{\text{V}}}{\text{O}}}_{2}$, mL min^−1^ kg^−1^), carbon dioxide output (${\dot{{\text{V}}}{\text{CO}}}_{2}$, L min^−1^) and ${\dot{{\text{V}}}{\text{E}}}$/${\dot{{\text{V}}}{\text{CO}}}_{2}$ slope were recorded or calculated. Since peak ${\dot{{\text{V}}}{\text{O}}}_{2}$ and power output (W) are influenced by age, sex, and body mass, percentages of predicted ${\dot{{\text{V}}}{\text{O}}}_{2peakth}$ (%${\dot{{\text{V}}}{\text{O}}}_{2peakth}$) and power output (%${\dot{{\text{W}}}}_{maxth}$) were also used to adjust study's analyses with regard to these parameters^22^. Theoretical maximum power output was obtained using the energy cost of a Watt, which averages 10.5 mlO_2_ W^−1^ on an electromagnetic ergocycle. The equation for predicting ${\dot{\text{V}}\text{O}}_{2peakth}$ was:

1 $${\dot{\text{V}}\text{O}}_{2peakth} \left( {{\text{ml}}\,{\text{kg}}^{ - 1} \,{\text{min}}^{ - 1} } \right) \, = \, 79.9 \, - \, \left( {0.39 \, \times \, age} \right) \, {-} \, \left( {13.7 \, \times \, gender \, \left[ {0 = male; \, 1 = female} \right]} \right) \, {-} \, (0.127 \, \times \, weight \, \left[ {lbs} \right]$$

The indexed arteriovenous oxygen difference or O_2_ extraction (AV-O_2_Δ in mL O_2_/L blood/m^2^ of body surface area) was calculated as the ratio of ${\dot{{\text{V}}}{\text{O}}}_{2}$ to cardiac index (CI) obtained from ${\dot{{\text{V}}}{\text{O}}}_{2}$ and echocardiography data recorded at each level of effort^23^. Preventive measures for the critical post-exercise period were applied and consisted in 2 min of active recovery followed by at least 8 min of passive recovery, including rehydration^5^. Patients were clinically monitored for 1 h following the procedure.

### Echocardiography evaluation

Comprehensive 2-dimensional, M-mode, Doppler and tissue Doppler echocardiography were performed according to contemporary guidelines by experienced sonographers blinded to medical record^24,25^. left ventricular (LV) end-diastolic volume, mass and left atrial volume were indexed to body surface area (LVEDV_ind_, LVM_ind_ and LAV_ind_, respectively). Complete echocardiography was performed at rest and LV contractility, cardiac output and diastolic function parameters were evaluated during sub-maximal exercise, at ∼ 2 mmol L^−1^ of blood lactate concentration, ∼ 4 mmol L^−1^ (BL4, just before exercise completion) and during the recovery phase. All studies were interpreted offline and in a completely blinded fashion by a cardiologist with extensive experience in resting and exercise echocardiographic assessment. Mitral inflow velocity pattern, peak velocities of early (E) and late (A) waves and E wave deceleration time (DT) were recorded. Tissue Doppler echocardiography was performed to measure early (e′) diastolic tissue velocities at the lateral mitral annulus, a witness of LV compliance. Lateral E/e′ ratio was used as a reflection of LV filling pressure (LVFP)^26^. Cardiac output (CO) was calculated by multiplying the LV systolic ejection volume (SEV) by the heart rate. SEV was measured by the time-velocity integral of aortic flow multiplied by the area of the ejection orifice. Cardiac index corresponded to the CO indexed to the body surface area. To evaluate cardiac adaptation during sub-maximal exercise, the ratios of values obtained at BL4 over baseline for CI, e′ and E/e′ were computed and analyzed^27–29^. All measures represent the mean of measurements from 3 beats for patients in sinus rhythm and the mean of 5 beats for the single patient in atrial fibrillation.

### Control population

A control population of healthy non-SCD subjects matched on age and gender and referred to the Physiology department for stress echocardiography was included. They performed stress echocardiography and blood lactate monitoring without gas exchange measurement. Hemodynamic and echocardiographic data were recorded at BL4 and used for comparison with the study population. Experimental conditions (location, sonographer, material for gas exchange and blood lactate measurement) were the same as the one used for the study population.

### Statistical analysis

The cohort was designed so that it could be divided into two groups based on the median of the occurrence of BL4 expressed as %${\dot{{\text{V}}}{\text{O}}}_{2peakth}$. As such, it enabled the evaluation of multiple variables associated with the earliness of reaching a blood lactate concentration of 4 mmol L^−1^ and explore diastolic cardiac adaptation impairment (defined in the “Echocardiography evaluation” section). Considering the observational nature of the study, regardless of any preconceived hypothesis and previous literature to build on, sample size calculations relied on the minimal magnitude of the effect sizes of both the differences between the two groups (based on BL4) but also the correlations between %${\dot{{\text{V}}}{\text{O}}}_{2peakth}$ at BL4 and patient’s characteristics. The inclusion of at least 26 subjects (i.e. 13 patients per group) allows the detection of statistically significant differences with a minimal effect size of 1 standard deviation, as well as the identification of Pearson correlation coefficients ≥ 0.5, with 80% power at 5% one-sided risk^30,31^. Descriptive results were expressed as mean (SD) or median [IQR] for continuous variables, and as numbers (%) for categorical variables. In accordance with our objective to associate cardiac diastolic impairment with early anaerobic shift, unadjusted group comparisons of continuous variables were performed using *t* tests or Mann–Whitney tests according to the normality of their distributions. Categorical variables were compared with the χ^2^ test. In addition to using our variable of interest (%${\dot{{\text{V}}}{\text{O}}}_{2peakth}$ at BL4) as a binary endpoint, we further analyzed it as a continuous variable using one-tailed Pearson correlation to evaluate relationships between BL4 occurrence with hemodynamic, cardiac respiratory and biological data. To identify factors independently associated with early BL4, multivariate analysis was then performed using binary logistic regression on parameters available in all the patient and significantly associated with or trending toward (p < 0.1) early vs. late BL4 occurrence (categorical variable based on the median of $\%{\dot{{\text{V}}}{\text{O}}}_{2peakth}$ at BL4). Notably, despite p < 0.1, documented PH and A wave on TTE were not included in the model since less than half of the patients had a RHC and A wave could not be measured in two patients due to AF or atrial hyperexcitability. Odds ratios (ORs) are presented with their 95% confidence intervals. A p value < 0.05 indicates statistical significance. Analyses were computed using SPSS 26 (IBM, Armonk, New York).

### Data sharing statement

For additional original data, please contact Thomas.dhumieres@aphp.fr.

## Results

### Population characteristics

Between September 2020 and July 2021, 30 SCA patients participated in the study. Twenty-nine patients reached 4 mmol L^−1^ of [lactate]_b_ during sub-maximal exercise and were included in the analysis. One patient had to stop exercise prematurely because of osteoarticular pain and was further excluded from the analysis. The baseline clinical, echocardiography and laboratory characteristics are summarized in Table 1. Briefly, mean age was 42 ± 12 years and 52% were men. Patients had frequent organ involvement (pulmonary hypertension, cerebral vasculopathy, chronic kidney disease, Table 1). Most of them (72%) were treated with hydroxyurea and 35% were included in an erythrocytapheresis program. Left ventricular ejection fraction was normal (LVEF = 62 ± 5%) for all patients. Left cardiac cavities were dilated (LVEDV_ind_ = 76 ± 16 mL m^−2^ and LAV_ind_ = 48 ± 26 mL m^−2^). Right ventricular function was preserved (TAPSE = 28 ± 5 mm). One patient was in atrial fibrillation. No sign of pulmonary obstructive disease was observed on the spirometry.

**Table 1:** Patients characteristics at Baseline.

Characteristics	Patients (n = 29)
Demographic and clinical characteristics	
Age (years)	41.5 ± 11.6
Male gender, n (%)	15 (51.7)
Body mass index (kg m^−2^)	23.4 ± 3.9
Systolic arterial pressure (mmHg)	134 ± 19.1
Diastolic arterial pressure (mmHg)	82.1 ± 12.7
Mean arterial pressure (mmHg)	99.5 ± 14.1
Heart rate (bpm)	77.8 ± 14.5
Oxygen saturation (%)	96.7 ± 2.82
Treatments	
Hydroxyurea, n (%)	21 (72.4)
Erythrapheresis, n (%)	10 (34.5)
Beta-blocker, n (%)	9 (31)
Events and organ damage	
Occurrence of VOC and/or ATS, n (%)	28 (96.5)
Intensive care unit admission, n (%)	11 (37.9)
Pulmonary hypertension on RHC (n = 14), n (%)	7 (50)
Cerebral vasculopathy, n (%)	10 (34.5)
Retinopathy, n (%)	24 (82.8)
Kidney damage without CKD*, n (%)	8 (27.6)
Kidney damage with CKD**, n (%)	7 (24.1)
Skin ulcers, n (%)	6 (20.7)
Osteonecrosis, n (%)	9 (31.0)
Priapism, n (%)	9 (60.0)
Biology	
Hemoglobin (g dL^−1^)	8.7 ± 1.1
Leukocytes (G L^−1^)	7.9 ± 2.8
Platelets (G L^−1^)	287 ± 179
Urea (mmol L^−1^)	5.3 ± 3.6
Creatinine (µmol L^−1^)	68.5 [46.7; 90.2]
Lactate dehydrogenase (IU L^−1^)	490 ± 188
Total bilirubin (µmol L^−1^)	40.4 ± 23.7
Aspartate transaminase (IU L^−1^)	43.3 ± 16.7
Alanine transaminase (IU L^−1^)	24.9 ± 9.6
Gamma-glutamyl transpeptidase (IU L^−1^)	105 ± 115
Alkaline phosphatase (IU L^−1^)	96 ± 53.3
Echocardiography	
LVEF (%)	61.7 ± 5.2
LVEDV_ind_ (mL m^−2^)	76.3 ± 16.4
LVM_ind_ (g m^−2^)	93 ± 23
GLS (%)	− 19 ± 3
Cardiac index (L min^−1^ m^−2^)	3.9 ± 0.7
E (cm s^−1^)	92 ± 23
E deceleration time (ms)	200 ± 46
A (cm s^−1^)	66 ± 21
E/A	1.5 ± 0.6
e′ lateral (cm s^−1^)	13.1 ± 3.1
E/e′	7.3 ± 2.1
LAV_ind_ (mL m^−2^)	57 ± 26
TAPSE (mm)	27.7 ± 5.2
Sdti (cm s^−1^)	14.3 ± 3
TRV (m s^−1^)	2.5 ± 0.4
Pulmonary acceleration time (ms)	122 ± 28
Mild to moderate valve disease (grade 2–3), n (%)	2 (7)
Ischemic heart disease, n (%)	1 (3.4)
Spirometry	
EV1 (L)	2.5 ± 0.7
FEV1/FVC	82.7 ± 6.7

Continuous data are presented mean ± SD or median [IQR] and nominal data in absolute value (%), *VOC* vaso-occlusice crisis, *ATS* acute thoracic syndrome, *RHC* right heart catheterization, *CKD* chronic kidney disease, *LVEF* left ventricular ejection fraction, *LVM*_*ind*_ left ventricular mass indexed to body surface, *LVED*_*ind*_ left ventricular end-diastolic volume indexed to body surface, *GLS* global longitudinal strain, *E* mitral inflow early filling velocity, *A* mitral inflow late filling velocity, *e′* lateral early diastolic myocardial velocity, *LAV*_*ind*_ left atrial volume indexed on body surface, *TAPSE* tricuspid annular plane systolic excursion, *Sdti* S wave using doppler tissu imaging of the tricuspid annulus, *TRV* tricuspid regurgitation velocity, *FEV1* forced expiratory volume in 1 s, *FVC* forced vital capacity. *tubulopathy and acidosis, proteinuria, glomerular hyperfiltration, hematuria. **GFR (CKD-EPI) < 60 mL/min/m^2^, or renal transplantation.

### Exercise responses and physical capacity indexes

**Figure 2:**
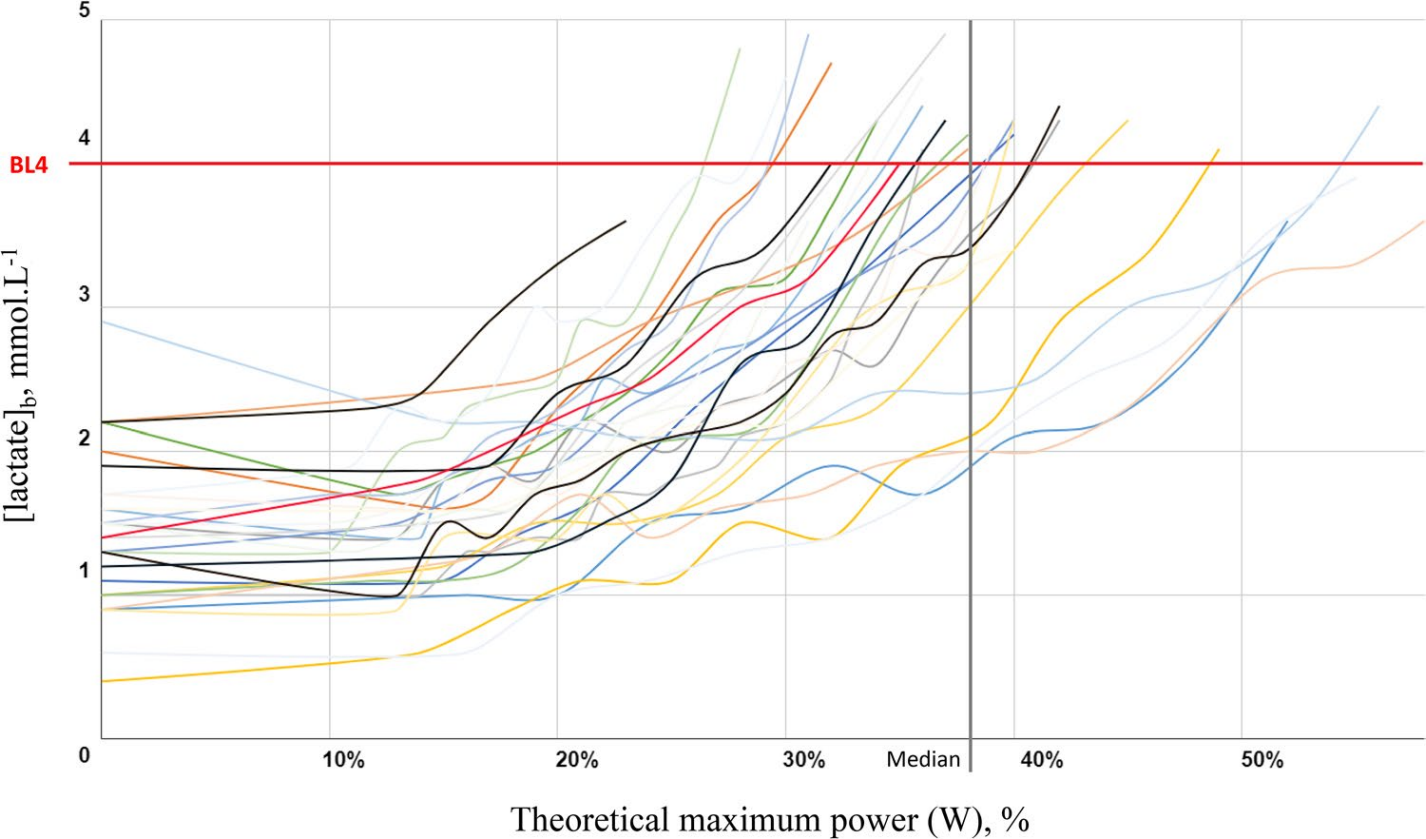
Blood lactate concentration vs. exercise load curves. Each curve represents a patient. The effort load is expressed in % of the maximum theoretical value.

Baseline lactatemia was 1.4 ± 0.5 mmol L^−1^ and exercise was stopped at 4.1 ± 0.1 mmol L^−1^. Cardiopulmonary (including gas exchange), and absolute or normalized to baseline echocardiography and hemodynamic data at BL2 and BL4 are summarized in Table 2. There was a great variability in the power outputs reached at BL4, with values ranging from 23 to 58% of %${\dot{{\text{W}}}}_{maxth}$ adjusted over age, sex, and body weight (Fig. 2). As expected, SCA patients displayed significant exercise intolerance as testified by the low ${\dot{{\text{V}}}{\text{O}}}_{2}$ values at BL2 (10.7 ± 3.13 mL kg^−1^ min^−1^, corresponding to 29.8 ± 7.9% of %${\dot{{\text{V}}}{\text{O}}}_{2peakth}$) and BL4 (14.6 ± 3.4 mL kg^−1^ min^−1^, 40.5 ± 8.5% of %${\dot{{\text{V}}}{\text{O}}}_{2peakth}$) as well as the corresponding power output at BL4 (71.2 ± 20.7 W, corresponding to 38.1 ± 9.0% of %${\dot{{\text{W}}}}_{maxth}$). For the reader's interest, detailed individual data on respiratory and hemodynamic parameters at BL2 and BL4 are provided in supplemental data.

**Table 2:** Evolution of clinical, echocardiographic, and cardiorespiratory parameters through sub-maximal exercise.

Exercise parameters	Values at BL2	Values at BL4
Power (W), (%${\dot{{\text{W}}}}_{{\text{maxth}}}$)	43.3 ± 15.5 (7.8 ± 2.6)	71.2 ± 20.7 (38.1 ± 9.0)
${\dot{{\text{V}}}{\text{O}}}_{2}$ (mL kg^−1^ min^−1^), (%${\dot{{\text{V}}}{\text{O}}}_{2{\text{peakth}}}$)	10.7 ± 3.13 (29.8 ± 7.9)	14.6 ± 3.4 (40.5 ± 8.5)
EV (L)	25.2 ± 6.7	41.5 ± 10.8
Ventilation reserve (%)	–	50 ± 12
$\dot{{\text{V}}}{\text{E}}$/ ${\dot{{\text{V}}}{\text{CO}}}_{2}$ slope	–	35.5 ± 7.4
Cardiac index (L min^−1^ m^−2^)	6.7 ± 1.7	7.8 ± 2.1
e′ (cm s^−1^)	17.6 ± 4.7	19.7 ± 4.7
E/e′	8.2 ± 2.4	7.9 ± 2.4
AV-O_2_Δ (mL L^−1^ m^−2^)	109 ± 32.7	131 ± 50
MAP (mmHg)	109 ± 18.4	117 ± 15
HR (bpm), (%HR_maxth_)	120 ± 21.2, (67.1 ± 12.4)	135 ± 26, (76.3 ± 15.8)
Oxygen saturation (%)	95.7 ± 3.6	95.6 ± 4.4
MET	–	4.2 ± 1

Data are presented mean ± SD. *BL2* blood lactate concentration of 2 mmol/l, *BL4* blood lactate concentration of 4 mmol/l, *%*${\dot{W}}_{maxth}$ power output expressed as % of the maximum predicted, $\dot{V}E$ ventilation rate, ${\dot{V}O}_{2}$ oxygen uptake, *%*${\dot{V}O}_{2peakth}$ VO_2_ expressed as % of the maximum predicted, *EV* extrapolated volume, ${\dot{V}CO}_{2}$ carbon dioxide uptake, *e′* lateral early diastolic myocardial velocity, *E* mitral inflow early filling velocity, *AV-O*_*2*_*Δ* arteriovenous oxygen difference, *MAP* mean arterial pressure, *HR* heart rate, *%HR*_*maxth*_ heart rate expressed as % of the maximum predicted, *MET* metabolic equivalent of task.

### Comparison with healthy non-SCD control group

Ten subjects composed the control group and were comparable to the SCA study population in terms of age and sex ratio (Supplemental data). Baseline mean arterial pressure was lower in the study population while cardiac index was higher (99 ± 14 vs. 111 ± 13 mmHg, p = 0.03 and 3.9 ± 0.7 vs. 2.7 ± 0.6 L min^−1^ m^−2^, p < 0.001 respectively). At BL4, power output expressed as % of the maximum predicted was much lower in the study population (38 ± 9 vs. 80 ± 24 of %${\dot{{\text{W}}}}_{maxth}$, p < 0.001). Diastolic adaptation at BL4 as assessed by the normalized to baseline e′ and E/e′, two key parameters of diastolic function was similar in the two groups, with a higher variability in the study population (Supplementary Data).

### Univariate analysis comparing two groups of SCA patients based on their %${\dot{V}O}_{2peakth}$ at BL4

The study population was then divided into two groups based on the median of the occurrence of BL4 expressed as %${\dot{{\text{V}}}{\text{O}}}_{2peakth}$ that was calculated at 39%. Fourteen patients constituted the lower exercise level group (BL4 < 39% group, %${\dot{{\text{V}}}{\text{O}}}_{2peakth}$= 34.4 ± 5.4%) and 15 patients were included in the upper exercise level group (BL4 ≥ 39% group, %${\dot{{\text{V}}}{\text{O}}}_{2peakth}$ = 46.2 ± 6.8%). Baseline clinical, biological and echocardiographic data of these two groups are reported in Table 3. In univariate analysis, hemoglobin (8.1 ± 1.1 vs. 9.2 ± 1.1 g dL^−1^, p = 0.009) and beta-blocker use [7 (50%) vs. 1 (13%), p = 0.03] were the only two baseline variables associated with early occurrence of BL4. None of the echocardiography data assessed at rest could discriminate the two groups. As expected, the BL4 < 39% group developed less power output and MET at BL4 than the BL4 ≥ 39% one (33 ± 4.7 vs. 42.8 ± 9.6% of ${\dot{{\text{W}}}}_{maxth}$, p = 0.002 and 3.6 ± 0.6 vs. 4.7 ± 1 MET, p = 0.003, respectively; Table 4). Notably, the BL4 < 39% group showed abnormal diastolic adaptation at BL4 as assessed by the normalized to baseline e′ and E/e′ (e′_BL4_/e′_rest_ = 1.4 ± 0.2 vs. 1.6 ± 0.3, p = 0.039 and E/e′_BL4_/ E/e′_rest_ = 1.2 ± 0.2 vs. 1.0 ± 0.2, p = 0.003). The BL4 < 39% group also had a trend for poorer peripheral oxygen extraction at BL4 (AV-O_2_Δ: 112 ± 33 vs. 147 ± 59 mL L m^−2^, p = 0.07) while increases in blood pressure, heart rate, and cardiac output were similar between the two groups (Table 4).

**Table 3:** Baseline clinical, biological and echocardiographic characteristics of patients depending on their oxygen uptake at BL4—Univariate analysis.

Characteristics	BL4% ${\dot{{\text{V}}}{\text{O}}}_{2{\text{peakth}}}$ < 39 (n = 14)	BL4% ${\dot{{\text{V}}}{\text{O}}}_{2{\text{peakth}}}$ ≥ 39 (n = 15)	p
Demographic and clinical characteristics			
Age (years old)	44 ± 10	38 ± 13	0.21
Male gender, n (%)	9 (64.3)	6 (40)	0.2
Body mass index (kg m^−2^)	22.9 ± 3.1	23.9 ± 4.7	0.37
Systolic arterial pressure (mmHg)	131 ± 18	137 ± 20	0.4
Diastolic arterial pressure (mmHg)	79 ± 10	85 ± 14	0.25
Heart rate (bpm)	73 ± 14	79 ± 13	0.23
Oxygen saturation (%)	95.6 ± 2.7	97.5 ± 2.7	0.07
Treatments			
Hydroxyurea, n (%)	11 (78.6)	10 (66.7)	0.5
Erythrapheresis, n (%)	5 (35.7)	5 (33.3)	1
Beta-blocker, n (%)	7 (50.0)	2 (13.3)	**0.03**
Events and organ damage			
Occurrence of VOC and/or ATS, n (%)	13 (93)	15 (100)	0.9
Intensive care unit admission, n (%)	7 (50)	4 (27)	0.2
Pulmonary hypertension on RHC (n = 14), n (%)	6 (60)	1 (25)	–
Cerebral vasculopathy, n (%)	5 (36)	5 (3)	0.8
Retinopathy, n (%)	11 (78)	13 (87)	0.9
Kidney damage without CKD*, n (%)	5 (36)	3 (20)	0.2
Kidney damage with CKD**, n (%)	5 (36)	2 (13)	0.2
Skin ulcers, n (%)	4 (29)	2 (13)	0.3
Osteonecrosis, n (%)	5 (36)	4 (27)	0.5
Priapism, n (%)	6 (67)	3 (50)	0.5
Biology			
Hemoglobin (g dL^−1^)	8.1 ± 1.1	9.2 ± 1.1	**0.007**
Leukocytes (/mm^3^)	7.2 ± 2.9	8.3 ± 2.6	0.32
Platelets (G L^−1^)	257 ± 159	316 ± 199	0.4
Lactate dehydrogenase (log)	2.7 ± 0.1	2.6 ± 0.2	0.15
Total bilirubin (µmol L^−1^)	45.6 ± 7.4	34.1 ± 13.6	0.17
Aspartate transaminase (IU L^−1^)	45.6 ± 19.2	38.7 ± 14.9	0.31
Resting blood lactate (mmol L^−1^)	1.5 ± 0.4	1.4 ± 0.7	0.56
Echocardiography			
LVEF (%)	61 ± 6	62 ± 5	0.43
LVEDV_ind_ (mL m^−2^)	79 ± 20	74 ± 12	0.37
LVM_ind_ (g m^−2^)	98 ± 30	89 ± 15	0.33
GLS (%)	− 19.2 ± 3.2	− 18.8 ± 4.1	0.73
Cardiac Index (L min^−1^ m^−2^)	3.9 ± 0.6	3.9 ± 0.8	0.83
E (cm s^−1^)	91 ± 30	93 ± 15	0.77
E deceleration time (ms)	213 ± 55	185 ± 29	0.11
A (cm s^−1^)	59 ± 20	73 ± 20	0.08
E/A	1.7 ± 0.8	1.3 ± 0.2	0.14
e′ lateral (cm s^−1^)	14 ± 3	13 ± 3	0.42
E/e′	6.8 ± 1.7	7.8 ± 2.4	0.18
LAV_ind_ (mL m^−2^)	62 ± 25	53 ± 27	0.35
TAPSE (mm)	27 ± 6	28 ± 4	0.56
TRV (m s^−1^)	2.6 ± 0.5	2.4 ± 0.4	0.23
Pulmonary acceleration time (ms)	120 ± 26	125 ± 31	0.63
Mild to moderate valve disease, n (%)	1	1	–
Ischemic heart disease, n (%)	0	1	–

Significant values are in bold.

Continuous data are presented mean ± SD and nominal data in absolute value (%), *VOC* vaso-occlusice crisis, *ATS* acute thoracic syndrom, *PH* pulmonary hypertension, *RHC* right heart catheterization, *CKD* chronic kidney disease, *LVEF* left ventricular ejection fraction, *LVM*_*ind*_ left ventricular mass indexed to body surface, *LVED*_*ind*_ left ventricular end-diastolic volume indexed to body surface, *GLS* global longitudinal strain, *E* mitral inflow early filling velocity, *A* mitral inflow late filling velocity, *e′* lateral early diastolic myocardial velocity, *LAV*_*ind*_ left atrial volume indexed on body surface, *TAPSE* tricuspid annular plane systolic excursion, *TRV* tricuspid regurgitation velocity. *tubulopathy and acidosis, proteinuria, glomerular hyperfiltration, hematuria. **GFR (CKD-EPI) < 60 mL/min/m^2^, or renal transplantation.

**Table 4:** Comparison of key exercise parameters of patients depending on their oxygen uptake at BL4—univariate analysis.

Exercise characteristics	BL4% ${\dot{{\text{V}}}{\text{O}}}_{2{\text{peakth}}}$ < 39 (n = 14)	BL4% ${\dot{{\text{V}}}{\text{O}}}_{2{\text{peakth}}}$ ≥ 39 (n = 15)	p
Values at BL4			
${\dot{{\text{V}}}{\text{O}}}_{2{\text{peakth}}}$(%)	34.4 ± 5.4	46.2 ± 6.8	** < 0.001**
${ \dot{{\text{W}}}}_{{\text{maxth}}}$(%)	33.0 ± 4.7	42.8 ± 9.6	**0.002**
AV-O_2_Δ (mL L^−1^ m^−2^)	112 ± 33	147 ± 59	0.07
${\mathrm{ HR}}_{{\text{maxth}}}$(%)	80.7 ± 11.9	82.5 ± 16.1	0.73
$\dot{\mathrm{ V}}{\text{E}}$/ ${\dot{{\text{V}}}{\text{CO}}}_{2}$ slope	36.7 ± 8.3	34.4 ± 6.5	0.42
Oxygen saturation (%)	94.5 ± 5.2	95.5 ± 5.4	0.62
MET	3.6 ± 0.6	4.7 ± 1.0	**0.003**
BL4/resting value ratio			
AV-O_2_Δ ratio	1.6 ± 0.2	1.7 ± 0.6	0.44
Cardiac index ratio	2.1 ± 0.3	2.1 ± 0.5	0.59
e′ ratio	1.4 ± 0.2	1.6 ± 0.3	**0.04**
E/e′ ratio	1.2 ± 0.2	1.0 ± 0.2	**0.003**
MAP ratio	1.2 ± 0.2	1.2 ± 0.1	0.70

Significant values are in bold.

Continuous data are presented mean ± SD. *BL4* blood lactate concentration of 4 mmol/l, ${\dot{V}O}_{2peakth}$ VO_2_ expressed as % of the maximum predicted, ${\dot{W}}_{maxth}$ power output expressed as % of the maximum predicted, *MET* metabolic equivalent of task, *AV-O*_*2*_*Δ* arteriovenous oxygen difference, *%HRmaxth* heart rate expressed as % of the maximum predicted, *e′* lateral early diastolic myocardial velocity, *E* mitral inflow early filling velocity, *MAP* mean arterial pressure.

### Multivariate analysis using logistic regression

Baseline hemoglobin, beta-blocker use, oxygen saturation at rest, AV-O_2_Δ at BL4, and BL4 to baseline ratio of e′ and E/e′ were entered into multivariate analysis (Table 5). Baseline hemoglobin (OR = 3.5 [1; 13], p = 0.05) and E/e′ ratio (OR = 10^–3^ [10^–4^; 0.6], p = 0.04) were independently associated with early occurrence of BL4.

**Table 5:** Multivariate analysis using logistic regression.

Parameters associated with early BL4	${\dot{{\text{V}}}{\text{O}}}_{2peakth}$< 39% (n = 14)	${\dot{{\text{V}}}{\text{O}}}_{2peakth}$≥ 39% (n = 15)	p-value in univariate analysis	p-value in multivariate analysis	OR (CI 95%)
Hemoglobin (g dL^−1^)	8.1 ± 1.1	9.2 ± 1.1	0.009	**0.05**	3.6 [1; 13]
E/e′ ratio	1.2 ± 0.2	1.0 ± 0.2	0.003	**0.04**	10^–3^ [10^–6^; 0.6]
e′ ratio	1.4 ± 0.2	1.6 ± 0.3	0.04	0.6	–
Beta-blocker, n (%)	7 (50.0)	2 (13.3)	0.03	0.2	–
AV-O_2_Δ* (mL L m^−2^)	112 ± 33	147 ± 59	0.07	0.7	–
Oxygen saturation (%) *at BL4	95.6 ± 2.7	97.5 ± 2.7	0.07		

Significant values are in bold.

### Correlations between %${\dot{V}O}_{2peakth}$ at BL4 and parameters of interest based on group comparison

Consistently with previous analysis, E/e′ and hemoglobin where the only parameters that showed moderate correlation to %${\dot{{\text{V}}}{\text{O}}}_{2peakth}$ at BL4 (r = 0.39, p = 0.02 and r = − 0.30, p = 0.05, respectively; supplemental data). Notably, correlations were also sought with all continuous variables presented in Table 1 along with BL4 hemodynamic and echocardiography data shown in Table 2. Total bilirubin level correlated with %${\dot{{\text{V}}}{\text{O}}}_{2peakth}$ at BL4 (r = − 0,33, p = 0.04) while no other parameter did (data not shown).

### Protocol safety

No severe clinical events occurred during the stress test. Electrocardiogram abnormalities during exercise were observed in 4 patients (two with transient ST segment depression, one with numerous premature ventricular complex and one with a short and non-sustained ventricular tachycardia of 8 complex). Arterialized blood gas analysis was performed for 21 patients two minutes after exercise completion and revealed a mean pH of 7.40 ± 0.04 associated with a [lactate]_b_ of 4.9 ± 0.6 mmol L^−1^. Blood acidosis with pH < 7.35 was observed for only one patient, who presented an end-stage kidney disease (Supplementary Data).

## Discussion

In this pilot study, we used an innovative and integrated approach which analyzes simultaneously hemodynamic, cardiac, respiratory, and metabolic parameters during stress test to highlight diastolic cardiac abnormalities linked to premature blood lactate accumulation. To our knowledge, this is the first application of “diastolic stress test” in conjunction with lactate monitoring in SCA patients. Our main findings were: (1) As hypothesized, impaired diastolic adaptation to exercise is independently associated with an early occurrence of BL4, such as low hemoglobin level. (2) Poor physical capacities are observed in all patients, thus reflecting their high vulnerability to exercise. Our protocol of sub-maximal stress test appears safe and feasible despite the severity of SCA patients.

### Stress-induced diastolic dysfunction is associated with poor exercise capacity and premature lactate elevation

**Figure 3:**
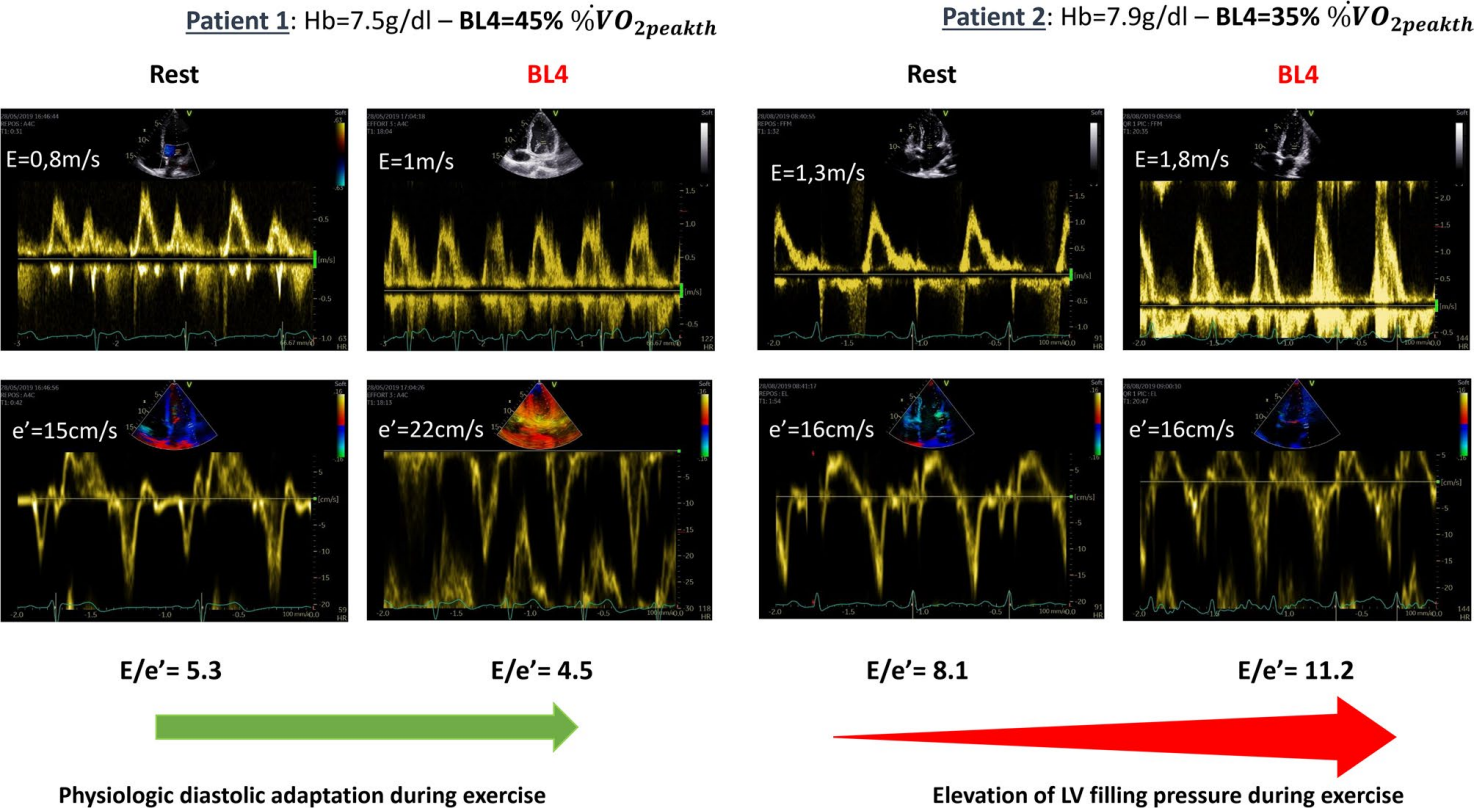
Representative example of physiological (Patient 1) vs. maladaptive (Patient 2) diastolic adaptation during exercise.

While baseline echocardiographic data were similar in the two groups (defined according to their %${\dot{{\text{V}}}{\text{O}}}_{2peakth}$ at BL4), the diastolic stress test unmasked poor diastolic adaptations that was independently associated with an early increase of [lactate]_b_ during a sub-maximal exercise. The evaluation of diastolic function during exercise is a new test, particularly useful to contribute to the challenging diagnosis of heart failure with preserved ejection fraction by identifying elevated LV filling pressures^26,27^. Yet, diastolic stress test and concomitant evaluation of [lactate]_b_ has not been evaluated in assessing cardiac function and vulnerability to exercise in SCA population. In our study, the increase in cardiac output was similar in both groups, but the increase in e′ velocity at BL4 was lower in the low physical capacity group (< 39% ${\dot{{\text{V}}}{\text{O}}}_{2peakth}$ at BL4), reflecting a subtle impairment in LV compliance. Indeed, a stress-induced increase in cardiac output associated with an altered LV compliance may be responsible for elevated LV filling pressures and alveolar edema. Accordingly, E/e′ ratio, a relevant index of LV filling pressure, increased in the BL4 < 39% group, indicative of a LVFP elevation^26^, while E/e′ ratio remained stable in the BL4 ≥ 39% group, reflecting a harmonious cardiac adaptation to exercise (Fig. 3). Our data extend previous reports on an independent association between E/e′ at rest and %${\dot{{\text{V}}}{\text{O}}}_{2peakth}$ or 6-min walk distance in SCA patients, suggestive of existing atrial cardiomyopathy^13,18,20^. However, we did not find such discriminant abnormalities on resting echocardiography, highlighting the challenge of diagnosing diastolic dysfunction in resting conditions in SCA population^15,16^. Thus, our results suggest that diastolic stress test during sub-maximal exercise is a valuable and safe tool in the early detection of subtle cardiac impairment in SCA patients.

Oxidative metabolism being directly limited by low oxygen transport capacities, our results also underline the expected role of anemia as a factor associated with the early occurrence of BL4^32^. Furthermore, the degree of anemia is mainly linked to the extent of hemolysis in SCA patients, which also alter peripheral oxygen exchanges through the development of systemic vasculopathy^33,34^. However, our study did not show any association between hemolysis parameters and BL4 occurrence. This could be explained by the small population size, but also by the lack of specificity of the routinely used hemolysis markers (LDH and bilirubin)^35^. Since innovative therapies increase (i) hemoglobin level by decreasing HbS sickling and erythrocytes hemolysis, and (ii) oxygen transport and supply, the evaluation of their impact on BL4 occurrence would be of particular interest^36–38^. Such therapeutic strategies could indeed delay the onset of BL4 and decrease complications associated with blood acidosis. In line with the latter hypothesis, sub-maximal exercise testing with early occurrence of BL4 could be the starting point of reinforced therapies.

### Poor exercise capacities and high metabolic vulnerability

Our study highlights the low physical capacities of SCA patients in relation to the poor transport, supply, extraction and utilization of oxygen at the muscular level^2,39–41^. It also adds mechanistic information, linking exercise diastolic dysfunction and anemia to the left-shift of [lactate]_b_ vs. work rate curve. The comparison with the control group in our study underlines the low power developed by SCA patients at equivalent metabolic stress levels (BL4). In the same way, healthy non-athlete adult individuals reach about 45% and 65% of their ${\dot{{\text{V}}}{\text{O}}}_{2max}$ at BL2 and BL4, respectively, while in the present study the corresponding values were 29.8 ± 7.9% and 40.5 ± 8.5%^42^. Furthermore, our results underline a great heterogeneity between patients in their physical capacities at BL4, with values that could vary by a factor of 3, independently of age, sex and body weight. Since BL4 is indicative of the onset of lactate unsteady state and constant accumulation, leading to blood acidosis, the practice of daily life physical activities (e.g. cleaning, shopping, walking the dog) could already be for some patients a too heavy effort at the origin of the deleterious cascade of erythrocyte falciformation, leading potentially to hemolytic and vaso-occlusive complications^9^. In support with the latter hypothesis, by translating exercise intensity at BL4 into metabolic equivalent of task (MET), 3 (10%) patients displayed MET < 3.0 (light intensity), 24 (83%) were [3.0; 6.0] (moderate intensity) and only 2 (7%) patients had MET ≥ 6.0 (vigorous intensity)^43^. To go beyond the simple assessment of exercise tolerance, the early occurrence of BL4 in response to low intensity training could be a new indicator of patient’s vulnerability, unmasking a lack of adaptation facing metabolic stress situations. Such findings may be extended to other stress conditions such as fever, dehydration, or altitude. This pilot study paves the way for larger studies with patients’ follow-up to investigate the interest of combined diastolic stress test and [lactate]_b_ monitoring as a tool to identify patients at risk and personalize recommendations in terms of physical activities^2^.

### A safe procedure within our cohort

This protocol of integrative sub-maximal exercise evaluation was feasible with valid measurements in all patients. Although maximal cardiopulmonary exercise appears to be a safe testing procedure in children and adults with SCD^44^, we, as previously described by our team, ensured the safety of the examination by stopping incremental exercise for each patient as soon as a [lactate]_b_ of 4 mmol L^−1^ was reached. This sub-maximal exercise prevents patients from being exposed to a metabolic risk situation (acidosis) and potential related complications^21,45^. Despite an unselected and severe population including patients with frequent organ damage, no patient presented any adverse clinical event during exercise or the recovery phase. In addition, no [lactate]_b_ value exceeding 7 mmol L^−1^ was observed in early phase of the recovery, a value associated with a risk of acid–base imbalance^46^. Consistently, exercise-induced blood acidosis was only found in one patient, with an end-stage renal disease.

### Strengths and limitations

One key methodological strength of the present study was the extemporaneous monitoring of [lactate]_b_ that allowed to analyze cardiac and ventilatory parameters at homogeneous and comparable levels of metabolic stress between patients (from baseline to 4 mmol L^−1^). Indeed, the analysis at maximum effort involves many confounding factors such as joint and muscle pain or the willingness of patients that could mislead results. In addition, to evaluate the earliness of BL4, we expressed it in %${\dot{{\text{V}}}{\text{O}}}_{2peakth}$ in order to automatically adjust for age, sex and BMI and to allow a more relevant comparison between patients^47^. Our study is limited by its small sample size as well as the heterogeneity of the population, partly counterbalanced by the quality of the phenotyping and the homogenization of the metabolic stress level by blood lactate monitoring. Since pulmonary pressure is strongly associated with patient prognosis in SCD, TRV at BL4 would have been an interesting variable to potentially unmask exercise PH, but it was uninterpretable in more than half of the patients due to the difficulty of obtaining a flow of sufficient quality through examination^48,49^. Notably, beta-blocker regimen was more often administered to the group with the lowest exercise tolerance. This could reflect more frequent heart disease (such as post-capillary PH), or symptoms justifying the use of beta-blocker by the physician. However, given that the pathophysiology of sickle cell heart disease is rather restrictive added to the anemia and peripheral exchanges alteration, slowing down the heart rate could be deleterious^50,51^. With regards to the lack of evidence, beta-blockers should be cautiously used in SCA patients.

## Conclusion and perspectives

This pilot study provides a new understanding of the mechanisms associated with exercise limitation in SCA patients, adding the role of impaired cardiac diastolic function to anemia. Integrative, sub-maximal, lactate driven exercise evaluation could be safely used to stratify the severity of SCA patients by revealing latent heart impairment or significant metabolic vulnerability and could be the starting point for reinforced therapies and rehabilitation programs.

### Supplementary Information

Supplementary Information.

## Abbreviations

%${\dot{\text{V}}\text{O}}_{2peakth}$: Percentage of theoretical peak oxygen uptake
%${\dot{{\text{W}}}}_{maxth}$: Percentage of predicted power output
A: Peak late diastolic velocity
ACS: Acute chest syndrom
AV-O_2_Δ: Arteriovenous oxygen difference
BL4: 4 Mmol L^−^^1^ blood lactate concentration
CO: Cardiac output
CI: Cardiac index
CKD: Chronic kidney disease
CPET: Cardiopulmonary exercise test
DT: E Waves deceleration time
E: Peak early diastolic velocity
e′: Early diastolic velocity at the lateral site of the mitral annulus
ECG: Electrocardiogram
EV: Expired volume
FEV1: Forced expiratory volume in 1 s
FVC: Forced vital capacity
HbS: Hemoglobin S
HF: Heart failure
HR: Heart rate
LAV_ind_: Left atrium volume indexed to body surface area
LDH: Lactate dehydrogenase
LT1: First lactate threshold
LV: Left ventricle
LVEDV_nd_: Left ventricular end-diastolic volume indexed to body surface area
LVEF: Left ventricular ejection fraction
LVM_ind_: Left ventricular mass indexed to body surface area
MAP: Mean arterial pressure
MET: Metabolic equivalent of task
PH: Pulmonary hypertension
RHC: Right heart catheterization
SCA: Sickle cell anemia
SCD: Sickle cell disease
SEV: Systolic ejection volume
TAPSE: Tricuspid annular plane systolic anterior excursion
TTE: Transthoracic echocardiography
TRV: Tricuspid regurgitation velocity
$$\dot{{\text{V}}}{\text{E}}$$: Minute ventilation
${\dot{{\text{V}}}{\text{CO}}}_{2}$: Carbon dioxide output
$${\dot{{\text{V}}}{\text{O}}}_{2}$$: Oxygen uptake
W: Power output
