# Supplementary material for: Cardiac diastolic maladaptation is associated with the severity of exercise intolerance in sickle cell anemia patients
Source: Sci Rep. 2024 May 15;14:11095. doi: 10.1038/s41598-024-61689-w (PMC11096405; doi:10.1038/s41598-024-61689-w)
Supplement: Supplementary file 1 — Supplementary Information. [file 41598_2024_61689_MOESM1_ESM.docx]

**Supplemental data**

**Supplemental Table 1:** Individual exercise parameter from baseline to BL4

| **Patient** | **Age** | **Sex (men=1)** | **Weight (kg)** | **Height (m)** | **Exercice time** | **Power (W)** | **%Wmaxth** | **HR (bpm)** | **%HRmaxth** | **sAP (mmHg)** | **dAP (mmHg)** | **VO2 (ml/kg/min)** | **%VO2maxth** | **EV (L)** | **O2 sat (%)** |
| --- | --- | --- | --- | --- | --- | --- | --- | --- | --- | --- | --- | --- | --- | --- | --- |
| **1** |  |  |  |  | **Baseline** | 5 |  | 68 | 37,24 | 124 | 77 | 4,57 | 9,71 |  | 97 |
|  | 37 | 1 | 69,0 | 1,85 | **BL2** | 45 | 8,164 | 108 | 59,14 | 133 | 79 | 10,14 | 21,57 | 18,3 | 98 |
|  |  |  |  |  | **BL4** | 75 | 28,696 | 153 | 83,79 | 148 | 81 | 14,56 | 30,97 | 31,5 | 99 |
| **2** |  |  |  |  | **Baseline** | 5 |  | 70 | 38,16 | 123 | 62 | 6,12 | 12,82 |  | 100 |
|  | 37 | 1 | 62,0 | 1,78 | **BL2** | 60 | 11,751 | 108 | 58,87 | 148 | 84 | 17,72 | 37,12 | 34 | 100 |
|  |  |  |  |  | **BL4** | 95 | 39,955 | 125 | 68,14 | 141 | 84 | 21,32 | 44,67 | 48 | 100 |
| **3** |  |  |  |  | **Baseline** | 5 |  | 84 | 42,13 | 146 | 87 | 5,57 | 11,02 |  | 99 |
|  | 21 | 1 | 72,0 | 1,80 | **BL2** | 70 | 12,311 | 112 | 56,18 | 163 | 86 | 16,15 | 31,96 | 31 | 97 |
|  |  |  |  |  | **BL4** | 115 | 38,583 | 143 | 71,73 | 156 | 98 | 22,22 | 43,97 | 41,5 | 98 |
| **4** |  |  |  |  | **Baseline** | 5 |  | 89 | 49,72 | 123 | 81 | 5,42 | 17,45 |  | 99 |
|  | 41 | 2 | 57,0 | 1,56 | **BL2** | 40 | 8,306 | 131 | 73,18 | 133 | 81 | 13,14 | 42,32 | 25,5 |  |
|  |  |  |  |  | **BL4** | 65 | 51,978 | 174 | 97,20 | 174 | 92 | 16,72 | 53,85 | 41 | 98 |
| **5** |  |  |  |  | **Baseline** | 5 |  | 76 | 38,36 | 155 | 88 | 5,55 | 11,08 |  | 100 |
|  | 22 | 1 | 77,0 | 1,86 | **BL2** | 85 | 14,224 | 125 | 63,09 | 180 | 98 | 15,93 | 31,82 | 33,5 | 98 |
|  |  |  |  |  | **BL4** | 115 | 36,372 | 146 | 73,69 | 170 | 103 | 20,61 | 41,18 | 47 | 97 |
| **6** |  |  |  |  | **Baseline** | 5 |  | 85 | 45,48 | 145 | 89 | 3,85 | 9,32 |  | 99 |
|  | 33 | 1 | 89,0 | 1,83 | **BL2** | 55 | 8,243 | 123 | 65,81 | 163 | 111 | 10,28 | 24,86 | 32,5 | 99 |
|  |  |  |  |  | **BL4** | 100 | 34,183 | 160 | 85,61 | 183 | 72 | 15,11 | 36,53 | 53 | 97 |
| **7** |  |  |  |  | **Baseline** | 5 |  | 96 | 55,15 | 158 | 99 | 5,21 | 15,20 |  | 99 |
|  | 46 | 2 | 59,0 | 1,77 | **BL2** | 35 | 7,097 | 118 | 67,78 | 160 | 103 | 13,30 | 38,78 | 25,5 | 98 |
|  |  |  |  |  | **BL4** | 60 | 40,402 | 137 | 78,70 | 180 | 105 | 16,71 | 48,74 | 46 | 99 |
| **8** |  |  |  |  | **Baseline** | 5 |  | 99 | 51,24 | 156 | 106 | 7,82 | 19,12 |  | 99 |
|  | 27 | 2 | 43,0 | 1,57 | **BL2** | 40 | 9,990 | 150 | 77,64 | 167 | 100 | 17,16 | 41,95 | 28,5 | 97 |
|  |  |  |  |  | **BL4** | 55 | 41,689 | 175 | 90,58 | 168 | 106 | 20,43 | 49,94 | 40,5 | 98 |
| **9** |  |  |  |  | **Baseline** | 5 |  | 92 | 56,05 | 163 | 115 | 4,08 | 12,01 |  | 100 |
|  | 56 | 1 | 92,0 | 1,89 | **BL2** | 45 | 6,573 | 165 | 100,52 | 176 | 87 | 10,48 | 30,83 | 34,5 | 99 |
|  |  |  |  |  | **BL4** | 80 | 33,717 | 212 | 129,15 | 184 | 110 | 14,11 | 41,51 | 58 | 99 |
| **10** |  |  |  |  | **Baseline** | 5 |  | 72 | 37,60 | 119 | 75 | 4,84 | 14,45 |  | 93 |
|  | 29 | 2 | 70,0 | 1,65 | **BL2** | 20 | 3,591 | 101 | 52,75 | 119 | 75 | 7,37 | 21,99 | 17,5 | 90 |
|  |  |  |  |  | **BL4** | 40 | 23,021 | 141 | 73,64 | 119 | 75 | 13,27 | 39,59 | 36 | 84 |
| **11** |  |  |  |  | **Baseline** | 5 |  | 69 | 40,47 | 138 | 85 | 3,75 | 13,64 |  | 94 |
|  | 50 | 2 | 70,0 | 1,70 | **BL2** | 40 | 7,181 | 101 | 59,24 | 166 | 85 | 9,34 | 33,93 | 20 | 87 |
|  |  |  |  |  | **BL4** | 75 | 56,425 | 133 | 78,01 | 198 | 89 | 12,71 | 46,18 | 36,5 | 94 |
| **12** |  |  |  |  | **Baseline** | 5 |  | 90 | 49,92 | 98 | 58 | 4,55 | 12,78 |  | 97 |
|  | 40 | 2 | 55,0 | 1,69 | **BL2** | 25 | 5,319 | 114 | 63,24 | 98 | 58 | 8,79 | 24,67 | 19 | 97 |
|  |  |  |  |  | **BL4** | 50 | 34,568 | 145 | 80,43 | 134 | 79 | 12,75 | 35,77 | 31,5 | 98 |
| **13** |  |  |  |  | **Baseline** | 5 |  | 52 | 32,79 | 139 | 78 | 5,02 | 12,67 |  | 99 |
|  | 61 | 1 | 57,0 | 1,74 | **BL2** | 30 | 6,229 | 83 | 52,34 | 133 | 80 | 8,31 | 20,97 | 16,75 | 99 |
|  |  |  |  |  | **BL4** | 50 | 28,992 | 109 | 68,74 | 133 | 80 | 15,18 | 38,31 | 33,5 | 98 |
| **14** |  |  |  |  | **Baseline** | 5 |  | 85 | 51,75 | 159 | 88 | 4,43 | 15,94 |  |  |
|  | 56 | 2 | 56,0 | 1,62 | **BL2** | 20 | 4,203 | 104 | 63,32 | 157 | 59 | 6,41 | 23,05 | 14,5 | 97 |
|  |  |  |  |  | **BL4** | 40 | 38,084 | 115 | 70,02 | 200 | 105 | 10,79 | 38,81 | 25,5 | 95 |
| **15** |  |  |  |  | **Baseline** | 5 |  | 75 | 41,16 | 143 | 92 | 3,05 | 12,70 |  | 96 |
|  | 38 | 2 | 93,0 | 1,70 | **BL2** | 30 | 4,345 | 105 | 57,63 | 160 | 80 | 7,74 | 32,25 | 23 | 95 |
|  |  |  |  |  | **BL4** | 60 | 40,072 | 135 | 74,10 | 169 | 81 | 10,92 | 45,51 | 38 | 96 |
| **16** |  |  |  |  | **Baseline** | 5 |  | 75 | 43,77 | 153 | 88 | 1,79 | 4,71 |  | 95 |
|  | 49 | 1 | 77,0 | 1,76 | **BL2** | 65 | 10,877 | 127 | 74,11 | 247 | 136 | 5,86 | 15,41 | 32 | 96 |
|  |  |  |  |  | **BL4** | 90 | 39,796 | 145 | 84,61 | 246 | 112 | 7,47 | 19,64 | 45 | 95 |
| **17** |  |  |  |  | **Baseline** | 5 |  | 63 | 34,36 | 109 | 78 | 4,08 | 11,15 |  | 95 |
|  | 37 | 1 | 98,0 | 1,81 | **BL2** | 60 | 8,340 | 92 | 50,17 | 154 | 75 | 10,61 | 28,99 | 39 | 95 |
|  |  |  |  |  | **BL4** | 85 | 30,525 | 104 | 56,72 | 187 | 70 | 13,67 | 37,35 | 76 | 98 |
| **18** |  |  |  |  | **Baseline** | 5 |  | 61 | 33,84 | 127 | 81 | 2,89 | 7,20 |  | 98 |
|  | 40 | 1 | 75,0 | 1,70 | **BL2** | 55 | 9,386 | 93 | 51,59 | 148 | 83 | 10,07 | 25,05 | 26 | 98 |
|  |  |  |  |  | **BL4** | 95 | 40,298 | 118 | 65,45 | 160 | 93 | 16,08 | 40,01 | 46 | 97 |
| **19** |  |  |  |  | **Baseline** | 5 |  | 56 | 32,69 | 121 | 61 | 4,02 | 9,91 |  | 92 |
|  | 49 | 1 | 62,0 | 1,67 | **BL2** | 35 | 6,855 | 101 | 58,96 | 183 | 57 | 10,98 | 27,10 | 21 | 91 |
|  |  |  |  |  | **BL4** | 65 | 33,430 | 146 | 85,24 | 238 | 67 | 14,92 | 36,80 | 40 | 90 |
| **20** |  |  |  |  | **Baseline** | 5 |  | 84 | 45,45 | 104 | 64 | 3,80 | 16,09 |  | 93 |
|  | 35 | 2 | 93,0 | 1,65 | **BL2** | 55 | 7,966 | 123 | 66,55 | 139 | 79 | 11,31 | 47,97 | 38 | 95 |
|  |  |  |  |  | **BL4** | 85 | 58,256 | 141 | 76,29 | 163 | 79 | 15,67 | 66,43 | 55 | 95 |
| **21** |  |  |  |  | **Baseline** | 5 |  | 85 | 48,55 | 132 | 83 | 3,42 | 10,63 |  | 93 |
|  | 45 | 2 | 59,0 | 1,68 | **BL2** | 35 | 7,097 | 135 | 77,11 | 142 | 117 | 9,91 | 30,74 | 21 | 92 |
|  |  |  |  |  | **BL4** | 55 | 40,236 | 151 | 86,25 | 148 | 110 | 12,03 | 37,35 | 25,5 | 94 |
| **22** |  |  |  |  | **Baseline** | 5 |  | 55 | 33,23 | 144 | 92 | 3,92 | 14,91 |  | 92 |
|  | 54 | 2 | 62,0 | 1,62 | **BL2** | 20 | 3,917 | 71 | 42,89 | 144 | 92 | 7,16 | 27,25 | 15 | 91 |
|  |  |  |  |  | **BL4** | 35 | 32,220 | 88 | 53,16 | 144 | 92 | 9,99 | 38,02 | 24,5 | 82 |
| **23** |  |  |  |  | **Baseline** | 5 |  | 80 | 45,75 | 153 | 89 | 4,56 | 11,21 |  | 96 |
|  | 45 | 1 | 84,0 | 1,90 | **BL2** | 30 | 4,701 | 103 | 58,90 | 153 | 89 | 7,55 | 18,56 | 22 | 90 |
|  |  |  |  |  | **BL4** | 75 | 27,804 | 145 | 82,92 | 163 | 71 | 14,89 | 36,60 | 49 | 92 |
| **24** |  |  |  |  | **Baseline** | 5 |  | 87 | 45,41 | 109 | 75 | 3,59 | 10,86 |  | 100 |
|  | 28 | 2 | 70,0 | 1,63 | **BL2** | 40 | 7,181 | 126 | 65,76 | 132 | 70 | 9,31 | 28,18 | 22 | 100 |
|  |  |  |  |  | **BL4** | 65 | 38,144 | 154 | 80,37 | 134 | 98 | 12,19 | 36,89 | 31,5 | 100 |
| **25** |  |  |  |  | **Baseline** | 5 |  | 55 | 31,91 | 160 | 78 | 3,83 | 14,23 |  | 94 |
|  | 48 | 2 | 69,0 | 1,64 | **BL2** | 50 | 9,071 | 113 | 65,56 | 177 | 93 | 10,73 | 39,91 | 26 | 94 |
|  |  |  |  |  | **BL4** | 70 | 55,276 | 129 | 74,85 | 201 | 106 | 12,22 | 45,43 | 39,5 | 94 |
| **26** |  |  |  |  | **Baseline** | 5 |  | 61 | 32,45 | 122 | 72 | 3,28 | 6,82 |  | 94 |
|  | 32 | 1 | 78,0 | 1,93 | **BL2** | 40 | 6,629 | 94 | 50,01 | 118 | 79 | 9,10 | 18,93 | 22,5 | 94 |
|  |  |  |  |  | **BL4** | 80 | 26,210 | 116 | 61,72 | 169 | 76 | 13,13 | 27,34 | 47,5 | 94 |
| **27** |  |  |  |  | **Baseline** | 5 |  | 77 | 40,49 | 104 | 73 | 5,22 | 13,70 |  | 99 |
|  | 30 | 2 | 50,0 | 1,59 | **BL2** | 50 | 11,338 | 116 | 61,00 | 120 | 80 | 13,84 | 36,36 | 24 | 99 |
|  |  |  |  |  | **BL4** | 70 | 49,302 | 134 | 70,47 | 141 | 84 | 16,43 | 43,17 | 39 | 99 |
| **28** |  |  |  |  | **Baseline** | 5 |  | 84 | 50,76 | 128 | 81 | 4,95 | 13,15 |  | 96 |
|  | 55 | 1 | 71,0 | 1,75 | **BL2** | 40 | 7,107 | 122 | 73,72 | 148 | 87 | 10,59 | 28,11 | 24 | 95 |
|  |  |  |  |  | **BL4** | 60 | 29,260 | 161 | 97,28 | 167 | 86 | 11,70 | 31,05 | 35,5 | 90 |
| **29** |  |  |  |  | **Baseline** | 5 |  | 90 | 58,31 | 140 | 85 | 5,10 | 14,14 |  | 100 |
|  | 66 | 1 | 60,0 | 1,70 | **BL2** | 40 | 8,016 | 126 | 81,64 | 180 | 98 | 11,66 | 32,32 | 24,5 | 100 |
|  |  |  |  |  | **BL4** | 60 | 37,112 | 145 | 93,95 | 198 | 106 | 15,38 | 42,65 | 41 | 97 |

**Supplemental Table 2:** Comparison between control group and study population

| **Baseline and exercise characteristics** | **Control (n = 10)** | **Study population (n = 29)** | **p** |
| --- | --- | --- | --- |
| **Values at baseline**  Age (year)  Sex (female, n (%))  HR (bpm)  MAP (mmHg)  E/e’  CI (L.min^-1^.m^-2^)  **Values at BL4**  $\dot{W}_{maxth}$ (%)  HR_maxth_ (%)  Oxygen saturation (%)  MAP (mmHg)  E/e’  CI (L.min^-1^.m^-2^) | 46 ± 13  5 (50)  82 ± 9  111 ± 13  5.8 ± 1.4  2.7 ± 0.6  **80 ± 24**  86 ± 8  96.1 ± 1.4  131 ± 17  6.6 ± 1.7  7.0 ± 1.7 | 42 ± 12  14 (48)  76 ± 13  99 ± 14  7.3 ± 2.1  3.9 ± 0.7  **38 ± 9**  82 ± 14  95.6 ± 4.3  117 ± 15  7.9 ± 2.4  8.1 ± 1.6 | 0.3  0.9  0.2  **0.03**  **0.04**  **<0.001**  **<0.001**  0.35  0.74  **0.02**  0.1  0.07 |
|  |  |  |  |
|  |  |  |  |
|  |  |  |  |
|  |  |  |  |
|  |  |  |  |
| **BL4/resting value ratio**  MAP ratio  Cardiac index ratio  e’ ratio  E/e’ ratio | 1.2 ± 0.1  2.7 ± 1.0  1.7 ± 0.2  1.1 ± 0.1 | 1.2 ± 0.2  2.1 ± 0.4  1.5 ± 0.3  1.1 ± 0.3 | 0.9  **0.02**  0.2  0.6 |
|  |  |  |  |
|  |  |  |  |
|  |  |  |  |
|  |  |  |  |
|  |  |  |  |

*Continuous data are presented mean±SD. BL4 indicates blood lactate concentration of 4mmol/l;* $\dot{W}_{maxth}$*,* *power output expressed as % of the maximum predicted; %HR_maxth_, heart rate expressed as % of the maximum predicted; e’, lateral early diastolic myocardial velocity; E, mitral inflow early filling velocity; CI, cardiac index MAP, mean arterial pressure.*

**Supplemental Table 3 :** One-tailed Pearson’s correlation between ${\dot{\mathbf{V}}\mathbf{O}}_{\mathbf{2}\boldsymbol{peakth}}$ at BL4 with baseline and exercise data

| **Correlation with %**${\dot{\mathbf{V}}\mathbf{O}}_{\boldsymbol{2}\boldsymbol{peakt}\boldsymbol{h}}$ **at BL4** | **r values** | **p** |
| --- | --- | --- |
| Hemoglobin (g.dL^-1^) | 0.39 | **0.02** |
| E/e’ ratio | -0.30 | **0.05** |
| e’ ratio | 0.2 | 0.1 |
| AV-O_2_Δ at BL4 (mL.L^-1^.m^-2^) | 0.3 | 0.06 |
| Oxygen saturation at rest (%) | 0.23 | 0.11 |

**Supplemental Table 4:** Clinical and biological characteristics of safety assessment during the examination and until the end of the monitoring period

| **Clinico-biological characteristics** | **Patients (n= 29)** |
| --- | --- |
| **Clinical events**  Clinical event, n (%)  ECG modification*, n (%) | 0 (0)  4 (13.8) |
|  |  |
|  |  |
| **Arterialized blood gas analysis** (n=21)**  pH  Metabolic acidosis with pH < 7.35, n (%)  CO2P (mmHg)  O2P (mmHg)  Lactate (mmol.L^-1^)  Base excess (mmol.L^-1^)  Bicarbonate (mmol.L^-1^) | 7.4 ± 0.04  1 (4)  33.1 ± 3.4  94.6 ± 15  4.9 ± 0.6  - 3.9 ± 2.7  21.1 ± 2.8 |
|  |  |
|  |  |
|  |  |
|  |  |
|  |  |
|  |  |
|  |  |

*Continuous data are presented mean±SD and nominal data in absolute value (%). CO2P indicates carbon dioxide pressure; O2P, oxygen pressure. *Two patients with sub-ST segment shift, one patient with a few premature ventricular contractions and one patient with a burst of 8 complex of non-sustained ventricular tachycardia.**Within 2 minutes after exercise completion.*
